# Cowpea legumin preservative impacts on beef ribeye and implications on antibiotic resistant food borne pathogens

**DOI:** 10.1038/s41538-024-00337-z

**Published:** 2024-11-19

**Authors:** Wesam Dawam, Shimaa Edris, Ali Osman, Mai Elsheikh, Ahmed Hamad, Mahmoud Sitohy, Islam Sabeq

**Affiliations:** 1 Department of Food Hygiene and Control, Faculty of Veterinary Medicine, Benha University, Tukh Qalyubia, Benha, 13736 Egypt; 2 Department of Biochemistry, Faculty of Agriculture, Zagazig University (ZU) Zagazig, Zagazig, 44519 Egypt

**Keywords:** Antimicrobial resistance, Medical humanities, Applied microbiology, Pathogens, Health humanities

## Abstract

The current study assessed the minimum inhibitory concentration (MIC) of Cowpea-legumin (*CPL*) against antibiotic-resistant foodborne pathogens (FBP), the consequences on *Longissimus thoracis et lumborum* (*LTL*) quality and shelf-life, and the growth curves of inoculated FBPs. Fresh *LTL*-steaks were enriched with either 0.5 mg/g (*CPL0.5*) or 1 mg/g (*CPL1*) and evaluated over 15 chilling-days at 4 °C. Antibiotic-resistant *Salmonella enterica* and *Escherichia coli* were inhibited by 25 and 3.125 mg/mL, respectively, while antibiotic-susceptible FBPs and antibiotic-resistant *Staphylococcus aureus* were suppressed by 0.1 mg/mL *CPL*. *CPL1*-fortification produced fully tender *LTL* that was initially yellower/less red than the control, then turned brighter red with storage. *CPL* demonstrated promising dose-dependent antioxidant, and antibacterial activities against native spoilage and antibiotic-resistant/susceptible FBPs. *CPL*’s proteinaceous composition, besides sample size, might impact stability. Conclusively, *CPL* demonstrated promising preservative stability in fresh meat for a maximum of fifteen-days and represents a viable antimicrobial alternative in battle against antibiotic-resistance.

## Introduction

Global consumption of beef proteins is expected to increase by 14% by 2030 compared to the base period average of 2018-2020, while beef protein availability is expected to increase by 5.9% by 2030^1^. Over the last few decades, population growth and urbanization have led to a rapid increase in the diverse types of waste, including solid organic waste (including protein) generated in people’s daily lives and industrial production. Hence by 2050, about 340 million tons of waste will accumulate, compared to 249 million tons in 2030, representing a 36.5% relative increase^2^.

Meat spoilage (MS) is a sophisticated process of microbial biodiversity that involves numerous distinct microbial interactions. The health of people is negatively impacted by MS, including the meat products waste along with significant losses in production, storage, transportation, and marketing^3^. Besides microbial spoilage, lipid and protein oxidation is the second most significant factor in meat deterioration in meat products. Meat palatability and other organoleptic qualities are generally affected with lipid and protein oxidation, and also diminish nutritional value, intrinsic and extrinsic quality, and other issues^4^. Controlling MS is primarily dependent on the development of novel technologies and tactics for the preservation of meat products, both of which rely on delaying the establishment and/or proliferation of natural flora. Among these approaches is the use of synthetic antioxidants and antimicrobials (e.g., nitrites, sulfites, benzoic acid, and sorbic acid) to extend the shelf life of meat and meat products and prevent meat spoilage caused by microbial, lipid, and protein oxidation^5^. Synthetic antioxidants and/or antimicrobials are recognized globally in commerce and, when used following the relevant regulations and the allowed dosage, are safe for consumers^6,7^. Meat enterprises have been driven to replace synthetic antioxidants and antimicrobials in meat systems with plant-derived antimicrobial agents due to the public’s belief that natural compounds are generally safer and healthier^8^.

Antimicrobial resistance is a worldwide public health problem affecting humans and animals. Antimicrobial-resistant bacteria (AMR) cause around 2.8 million human diseases and 700,000 deaths yearly. This figure might climb to 10 million by 2050 if AMR is not effectively addressed^9^. Antimicrobial misuse is the leading driver of resistant bacteria growth in the food and environment, particularly in low- and middle-income nations. AMR-bacteria transmission from animals and the environment via food may result in the spread of resistance genes among bacterial species as well as between animals, humans, and the environment^10,11^.

Furthermore, there has been an increase in antimicrobial-resistant (AMR) bacteria, including resistance to antimicrobials not licensed for use in veterinary medicine, which has been detected in meat products^12^. These issues have prompted various food safety authorities to fight antimicrobial resistance to achieve optimal human health and well-being while also being internally related to animal health and the environment (One Health) because humans and animals are both affected by the same bacteria and are treated with the same antimicrobials^13^. Concerns regarding AMR bacteria, in addition to microbiological hazards, are among the major reasons food safety must be ensured. Natural antimicrobial approaches have been experimentally tested, such as using bacteriophages, antimicrobial peptides and proteins and phytobiotics, such as essential oils or propolis ethanol extracts^14,15^.

Leguminosae seeds are high in biological value protein, carbohydrates, vitamins, and minerals and have been demonstrated to be natural sources of antioxidants, anti-inflammatory, and antibacterial substances^16^. Legume phytochemicals—which include proteins—have sparked a new line of investigations in the food sector, academia, and pharmaceutical enterprises to develop a new class of potentially valuable natural antibacterial and/or preservative components and extracts that could be used in innovative, clean-labeled products to address the threat of antibiotic resistance to public health^16,17^. Additionally, plant-based ingredients blending into traditional meat products is the meat processing industry’s main priority in minimizing the negative effects on the environment, public health, and animal welfare^18^. Cowpea seed proteins, like soybean seed proteins, predominantly comprise globulins, which account for more than 51% of total protein composition, while albumins account for about 45%^19^. Previous research found that fortifying minced beef with chickpea legumin^20^ and cowpea 7S & 11S globulin^14,21^ had promising antibacterial benefits.

Additional investigations on legumes will be necessary to broaden the comprehension of multiple important but still unresolved topics, including the main antimicrobial compounds, mechanisms of action, microbial inactivation kinetics produced in novel matrices, MIC and MBC, antimicrobial activity stability, and formulation assays using real food matrices^16^. Another critical concern is their impact on the technological characteristics of food during shelf-life.

The current study attempts to determine whether increased Cowpea-legumin (CPL) levels can extend beef shelf-life beyond previous research findings, or whether its antibacterial and antioxidant stability is influenced by its proteinaceous composition and decreases over time. Thus, the goals of the current study included determining the minimum antibacterial concentration of *CPL* against antibiotic-resistant/susceptible food-borne pathogens and assessing their influence on shelf-life, antioxidant stability, quality attributes, and growth curves of artificially inoculated food-borne pathogens in beef longissimus steaks.

## Results

**Fig. 1:**
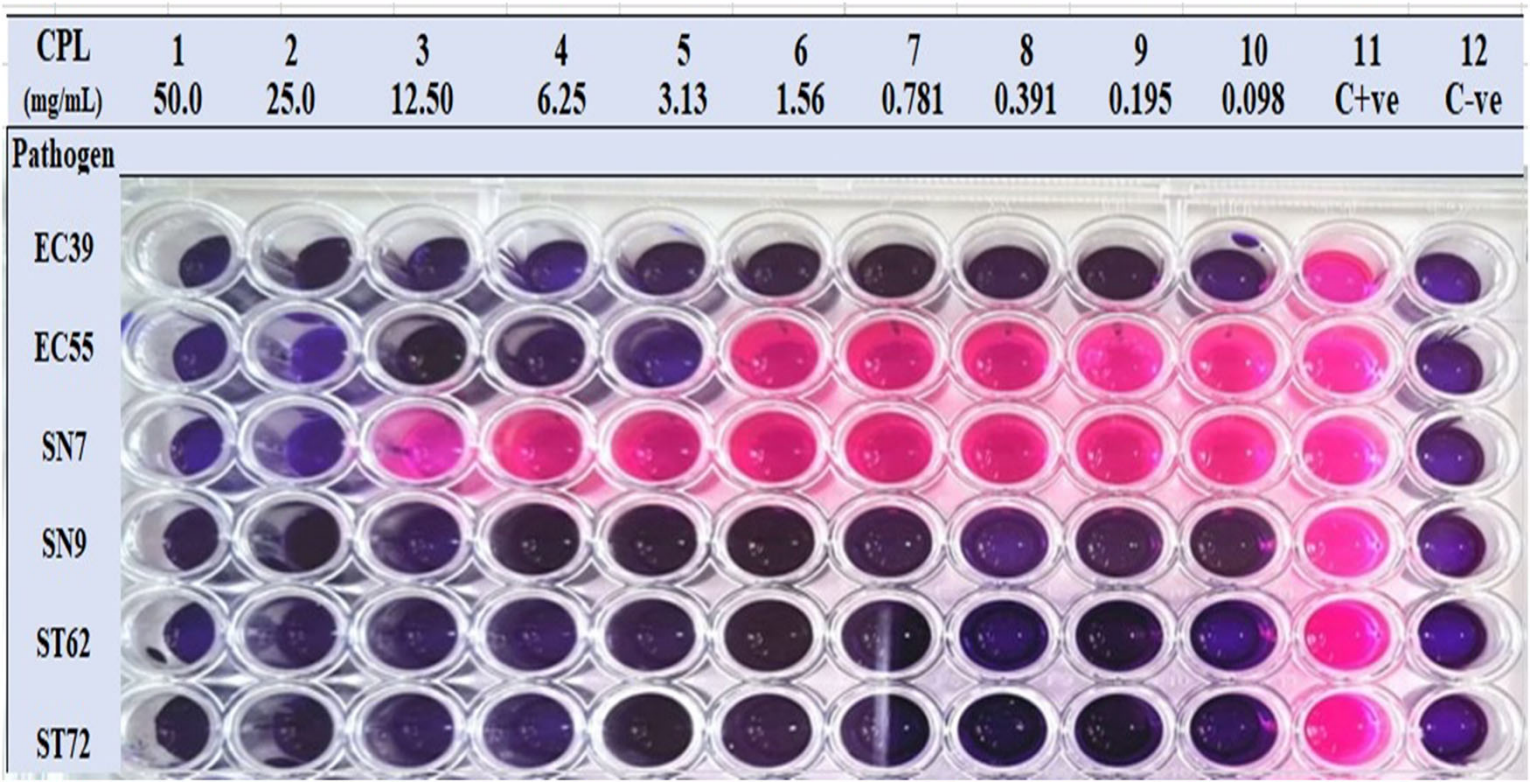
depicts the results of a resazurin-based turbidimetric microdilution method used to determine the minimum inhibitory concentration and biocidal effect of cowpea-legumin (*CPL*) on six antibiotic-susceptible and antibiotic-resistant pathogens, including two *Salmonella enterica* subspecies *enterica* (N7 and N9), two *Escherichia coli* (EC39, EC55), and two *Staphylococcus aureus* (ST62 and ST72). The isolates were classified as multidrug resistant (N7, EC55, and ST62) or non-resistant (EC39, N9, and ST72). All antibiotic-susceptible organisms and antibiotic-resistant *Staphylococcus aureus* were entirely eradicated by 0.098 mg/mL *CPL*; however, antibiotic-resistant *Salmonella enterica* and *E*. *coli* required higher biocidal doses of 50 and 6.25 mg/mL, respectively.

Figure 1 depicts the minimal inhibitory concentration and biocidal activity of cowpea-legumin (*CPL*) against antibiotic-susceptible and antibiotic-resistant FBPs. In a turbidimetric resazurin-based microdilution method, *CPL* at 0.098 mg/mL killed all antibiotic-susceptible (ST72) and antibiotic-resistant *Staphylococcus aureus* (ST62). Similar biocidal effects were observed in antibiotic-susceptible *Salmonella* and *E*. *Coli*; however, suppression of growth or death was observed in resistant *E*. *coli* (EC55) and *Salmonella* (N7) at higher *CPL* dosages of 6.5 and 50 mg/mL, respectively (Fig. 1).

Over 15 chilling days, Table 1 illustrates the impact of the *CPL* pretreatment on various physicochemical and oxidative stability parameters of *LTL*-steaks compared to the control. According to statistics, there was a substantial difference in the estimated physicochemical qualities of *LTL*-steaks due to the interaction between pretreatment with *CPL* and the storage period. WHC and drip loss among all estimated physicochemical qualities of *LTL*-steaks did not vary due to the interaction between the *CPL* pretreatment and chilling duration (Table 1). Meanwhile, at various monitoring points across each group, the length of chilling duration significantly impacted all estimated physicochemical characteristics of *LTL*-steaks. Similarly, except for WHC, redness (a*), and Chroma, all calculated physicochemical properties of *LTL*-steaks were significantly altered by *CPL* pretreatment. In contrast to the ascending trend of the control *LTL*-steaks pH, which reached the spoiling point 9-days post-treatment, *CPL* pretreatment demonstrated an up-and-down pattern, attaining poor pH quality at the 15^th^-day post-treatment (*p* < 0.05) (Table 1).

**Table 1:** Shows the impact of the cowpea-legumin pretreatment on various physicochemical and oxidative parameters of *Musculus longissimus thoracis* et *lumborum* (*LTL*)-steaks compared to the control over 15 chilling days

Parameters	Groups	Chilling points (day)						SEM^1^	*P* values		
		1	3	6	9	12	15		Time	*CPL*^*1*^	*CPL* *Time
pH	Control	6.05^Bd2^	6.21^Cd^	6.38^d^	7.1^Ac^	7.97^Ab^	8.5^Aa^	0.411	<0.001		
	*CPL1*^*1*^	6.22^Ae^	6.33^Ac^	6.16^f^	6.38^Bb^	6.29^Bd^	6.76^Ba^	0.086	<0.001		
	*CPL0.5*^*1*^	6.22^Ad^	6.28^Bc^	6.10^e^	6.35^Bb^	6.27^Bc^	6.57^Ca2^	0.064	<0.001		
	SEM	0.004	0.006	0.095	0.018	0.004	0.007				
	*p* value	<0.001	<0.001	0.452	<0.001	<0.001	<0.001		<0.001	<0.001	<0.001
WHC (%)^1^	Control	93.17^ab2^	88.10^b^	89.08^abB^	91.06^Aab^	80.50^c^	94.63^a2^	1.167	<0.001		
	*CPL1*	91.94	89.66	93.82^A^	89.21^B^	85.98	92.49	0.934	0.462		
	SEM	0.599	0.550	0.975	0.369	1.749	2.061				
	*p* value	0.22	0.218	0.027	0.046	0.136	0.274		<0.001	0.307	0.162
Purge loss (%)	Control	2.54^b^	2.26^bB^	4.15^ab^	4.24^ab^	5.67^ab^	7.58^a^	0.923	0.016		
	*CPL1*	2.89^d^	5.93^bcA^	5.57^c^	5.93^bc^	9.27^a^	8.09^ab^	0.481	<0.001		
	SEM	0.736	0.491	0.686	0.613	0.986	0.698				
	*p* value	0.748	0.002	0.198	0.110	0.087	0.714		<0.001	0.010	0.412
Cooking loss (%)	Control	40.87	41.34	40.05	38.22	36.63	46.04	1.315	0.188		
	*CPL1*	38.73^ab^	41.49^a^	40.15^ab^	37.16^b^	39.24^ab^	42.44^a^	0.782	0.019		
	SEM	0.616	1.091	0.499	0.532	0.912	2.993				
	*p* value	0.125	0.942	0.915	0.260	0.167	0.554		<0.001	0.001	<0.001
WBSF^1^	Control	8.51^aA2^	7.64^bA^	9.12^aA^	6.38^cA^	8.55^aA^	6.33^cA^	0.483	<0.001		
	*CPL1*	4.78^bcB^	4.62^cB^	4.36^cdB^	4.08^dB^	5.14^abB^	5.37^aB^	0.195	<0.001		
	SEM	0.109	0.205	0.149	0.110	0.155	0.218				
	*p* value	<0.001	<0.001	<0.001	<0.001	<0.001	0.016		<0.001	<0.001	<0.001
L*^1^	Control	40.4^aA^	39.4^abB^	40.4^a^	38.6^bcA^	39.0^bc^	38.1^cB^	0.381	<0.001		
	*CPL1*	39.5^cB^	41.6^bA^	40.24^c^	36.8^dB^	40.1^c^	44.3^aA^	1.016	<0.001		
	SEM	0.116	0.167	0.153	0.065	0.530	0.230				
	*p* value	0.012	0.001	0.687	<0.001	0.247	<0.001		<0.001	<0.001	<0.001
a*^1^	Control	17.2^aA2^	17.2^aA^	17^a^	15.6^bB^	15.4^bA^	15.1^bB^	0.404	<0.001		
	*CPL1*	16.0^cB^	14.5^dB^	16.6^bc^	17.2^abA^	14.5^dB^	17.3^aA2^	0.519	<0.001		
	SEM	0.139	0.112	0.162	0.211	0.185	0.095				
	*p* value	0.003	<0.001	0.196	0.009	0.031	<0.001		<0.001	0.293	<0.001
b*^1^	Control	2.5^cB2^	2.3^cdB^	4.1^a^	3.3^b^	2.0^dB^	2.01^dB^	0.345	<0.001		
	*CPL1*	3.1^bA^	3.3^bA^	3.4^b^	3.5^b^	3.0^bA^	5.2^aA2^	0.324	<0.001		
	SEM	0.148	0.079	0.183	0.123	0.122	0.035				
	*p* value	0.041	0.002	0.072	0.423	0.004	<0.001		<0.001	<0.001	<0.001
Chroma^1^	Control	17.4^aA^	17.3^aA^	17.5^a^	15.9^bB^	15.5^b^	15.2^bB^	0.424	<0.001		
	*CPL1*	16.3^cB^	14.8^dB^	16.9^b^	17.6^aA^	14.8^d^	18.1^aA^	0.558	<0.001		
	SEM	0.119	0.115	0.134	0.181	0.180	0.101				
	*p* value	0.003	<0.001	0.062	0.006	0.060	<0.001		.006	0.966	<0.001
Hue (*h*˚)^1^	Control	8.2^Bb^	7.7^Bb^	13.6^a^	12.1^a^	7.3^Bb^	7.6^Bb^	1.109	<0.001		
	*CPL1*	11.1^Ab^	12.9^Ab^	11.7^b^	11.5^b^	11.6^Ab^	16.6^Aa^	0.842	<0.001		
	SEM	0.550	0.281	0.700	0.566	0.463	0.060				
	*p* value	0.023	<0.001	0.166	0.470	0.003	<0.001		0.001	<0.001	<0.001
MDA (nM/g)^1^	Control	70.05^**b2**^	72.20^**b**^	79.52^**abA**^	92.55^**abA**^	92.80^**abA**^	113.96^**aA**^	7.506	0.024		
	*CPL1*	70.10	69.10	57.68^B^	55.17^B^	50.54^B^	55.37^B^	7.703	0.497		
	*CPL0.5*	69.7^b^	70.28^b^	68.23^AB^	73.49^AB^	71.31^AB^	83.63^AB2^	69.71	0.488		
	SEM	6.99	10.0	4.92	5.53	5.94	7.83				
	*p* values	0.999	0.977	0.054	0.019	0.007	0.008		0.149	0.000	0.031

^1^*CPL* Cowpea-legumin, *CPL1* 1 mg/g cowpea-legumin inclusion, *CPL0.5* 0.5 mg/g cowpea-legumin inclusion, *WHC* water holding capacity, *WBSF* Warner-Bratzler Shear Force, L* Lightness, a* redness, b* yellowness, Chroma color intensity, *h*˚ color saturation or hue angle, *MDA* Malondialdehyde, *SEM* standard error of the means.

^2^Different small letters within the row show significant changes across chilling times (*P* < 0.05), while different capital letters within the column indicate significant differences between treatments.

*LTL*-steaks pretreatment with *CPL* did not affect estimated WHC throughout the entire chilling period and always had similar values to control meat (*p* > 0.05), except for the sixth-day post-treatment, which had a greater WHC value than the control (*p* < 0.05) (Table 1). On the other hand, purge loss was impacted by the chilling storage interval in all groups, and an elevated loss pattern was associated with longer storage, peaking at the 15^th^ chilling-day, clearly in control. *CPL*-pretreatment displayed no effect on *LTL*-steaks purge loss (*p* > 0.05), which remained comparable to control across all chilling-points with the exception of the third chilling-day, when CPL-pretreated *LTL*-steaks had greater drip loss values (*p* < 0.05) (Table 1).

Compared to the control, *CPL1* pretreatment did not affect *LTL*-steaks cooking loss (CL) over the full experiment period (*p* > 0.05). The chilling interval demonstrated an effect on *CPL1*-pretreated *LTL*-steaks CL, but not on control; the lowest and highest CL occurred on the ninth and fifteenth chilling days, respectively (Table 1). Pretreatment of *LTL*-steaks with *CPL1* reduced WBSF values compared to controls, and extended chilling time had a significant effect on this feature in both groups (*p* > 0.05). Until the ninth day post-treatment, *CPL1* displayed a declining trend in the WBSF value (4.08) of *LTL*-steaks, but thereafter it increased dramatically (*p* > 0.05) (Table 1).

Except at the 6- and 12-day post-treatment, *CPL1* had a significant effect on *LTL*-steaks lightness (L*) compared to the control (*p* < 0.05). Furthermore, lengthy chilling storage significantly impacted this feature; whilst it decreased with longer storage of control *LTL*-steaks (40.4 at first day to 38.1 at 15 days), the treated *LTL*-steaks exhibited the highest L* on the final day post-treatment (*p* < 0.05). Throughout the entire storage period, pretreatment of *LTL*-steaks with CPL1 decreased redness (a*) and chroma compared to control (*p* < 0.05); however, on the 9-and 15-day post-treatment, it increased (*p* < 0.05). Additionally, longer storage significantly reduced the a* and chroma of the control *LTL*-steaks. In contrast, the treated *LTL*-steaks showed a roughly climbing trend, except for the 12- day post-treatment, which interestingly presented the lowest a* (14.5) and chroma (14.8) comparable to the 3- day (*p* < 0.05). Pretreatment of *LTL*-steaks with *CPL1* resulted in increased yellowness (b*) and hue (*h*˚) compared to the control group (*p* < 0.05), with comparable results at the sixth- and ninth-days post-treatment (*p* > 0.05). Extended chilling storage also had a substantial impact on b* and *h*˚ in the compared groups; whereas b* and *h*˚ of the control group showed a rising trend up to their highest values at the sixth- and ninth-day post-treatment, they thereafter showed a significant decline until the conclusion of the storage period (*p* > 0.05). CPL1 kept *LTL*-steaks b* and *h*˚ uniform throughout the chilling storage experiment but reached their peak at the 15-day post-treatment (*p* > 0.05) (Table 1).

Oxidation indicators revealed that both *CPL* pretreatment levels, *CPL1* and *CPL0.5*, greatly enhanced the *LTL*-steaks oxidative stability compared to the control, having a consistent effect throughout the whole storage period as opposed to the control’s noticeably rising curve. Also, the interaction between storage length and *CPL* pretreatment significantly affected oxidative stability (*p* < 0.05) (Table 1).

Table 2 illustrates the influence of the two *CPL* inclusion levels, *CPL1* and *CPL0.5*, on different *LTL*-steaks bacteriological indices and shelf-life compared to control over 15 chilling-days. Statistically, all *LTL*-steaks bacteriological indices were affected by the Cowpea-legumin pretreatment levels, storage interval, and their interaction (*p* < 0.05). Both *CPL* pretreatment levels, *CPL1* and *CPL0.5*, retarded all *LTL*-steaks bacteriological indices, APC, coliform, *Lactic acid bacteria* (LAB), and *Staphylococcus* count, of *LTL*-steaks from the first day compared to the control (*p* < 0.05). The *CPL* antimicrobial influence continued till the end of chilling storage at fifteen-day post-treatment (*p* < 0.05). Higher inhibition corresponded to higher *CPL* inclusion levels. Both *CPL* inclusion levels were able to retard APC below 6 logs CFU/g till the twelve-day post-treatment. The *CPL* antimicrobial effect was higher on *Staphylococcus* count than coliform, where higher inclusion levels retarded the *Staphylococcus* growth curve till the end of storage within the range of 2 to 2.6 log CFU/g. Also, significant differences were noticed between the six checkpoints of the growth curve of different estimated bacteriological indices of control and *CPL*-pretreated *LTL*-steak groups (*p* < 0.05). Also, the *LAB* growth curve was retarded by *CPL* inclusion levels till the fifteen-day post-treatment, with dose-dependent effects (*p* < 0.05).

**Table 2:** Shows the influence of the two Cowpea-legumin (*CPL*) inclusion levels on different *Musculus longissimus thoracis* et *lumborum* (*LTL*)-steaks bacteriological indices and shelf-life compared to control over 15 chilling days

Parameter	Groups	Chilling points (day)						SEM^1^	*P* values		
		1	3	6	9	12	15		Time	*CPL*^*1*^	*CPL* *Time
APC^1^	Control	4.86^Ac2^	5.15^Ac^	5.27^Ac^	7.09^Ab^	7.62^Ab^	8.29^Aa^	0.599	<0.001		
	*CPL1*^*1*^	4.37^ABcd^	3.13^Be^	3.87^Bd^	4.54^Bbc^	5.12^Bb^	6.31^Ba^	0.445	<0.001		
	*CPL0.5*^*1*^	3.70^Bd^	3.83^Bd^	4.08^Bd^	4.74^Bc^	5.49^Bb^	6.69^Ba2^	0.473	<0.001		
	SEM	0.129	0.168	0.073	0.149	0.082	0.138				
	*p* value	0.014	0.001	<0.001	<0.001	<0.001	<0.001		<0.001	<0.001	0.007
Coliform	Control	4.22^Ad2^	4.41^Ad^	4.91^Ac^	6.78^Ab^	7.77^Aa^	7.86^Aa^	0.685	<0.001		
	*CPL1*	2.99^Bc^	3.01^Cc^	2.69^Ccd^	3.19^Bc^	4.21^Cb^	5.72^Ba^	0.443	<0.001		
	*CPL0.5*	2.99^Bc^	3.41^Bb^	3.14^Bbc^	3.16^Bbc^	5.35^Ba^	5.65^Ba2^	0.524	<0.001		
	SEM	0.036	0.029	0.086	0.061	0.015	0.196				
	*p* value	<0.001	<0.001	<0.001	<0.001	<0.001	<0.001		<0.001	<0.001	<0.001
*LAB*^*1*^	Control	4.47^Ac2^	5.26^Ab^	5.64^Ab^	7.85^Aa^	7.54^Aa^	7.57^Aa^	0.589	<0.001		
	*CPL1*	4.09^Cb^	3.90^Bb^	3.60^Bb^	4.01^Cb^	5.35^Ba^	6.11^Ba^	0.404	<0.001		
	*CPL0.5*	4.16^Bbc^	3.94^Bcd^	3.69^Bd^	4.45^Bb^	5.57^Ba^	5.74^Ba2^	0.351	<0.001		
	SEM	0.012	0.151	0.097	0.069	0.199	0.142				
	*p* value	<0.001	0.001	<0.001	<0.001	0.001	0.002		<0.001	<0.001	<0.001
*Staphylococcus*	Control	3.67^d^	3.17^Ad^	3.58^Ad^	4.57^Ac^	5.31^Ab^	6.34^Aa^	0.494	<0.001		
	*CPL1*	3.74^a^	3.06^Ab^	2.0^Bc^	2.0^Cc^	2.2C^c^	2.0^Bc^	0.297	<0.001		
	*CPL0.5*	3.74^a^	2.26^Bcd^	2.0^Bd^	2.56^Bc^	3.24^Bb^	2.0^B^d	0.291	<0.001		
	SEM	0.051	0.079	0.017	0.069	0.157	0.007				
	*p* value	0.741	0.001	<0.001	<0.001	<0.001	<0.001		<0.001	<0.001	<0.001

^1^*CPL* Cowpea-legumin, *CPL1* 1 mg/g cowpea-legumin inclusion. *CPL0.5* 0.5 mg/g cowpea-legumin inclusion, *SEM* standard error of the mean; *APC* aerobic plate count, *LAB* Lactic acid bacteria.

^2^Different small letters within the row show significant changes across chilling times (*p* < 0.05), while different capital letters within the column indicate significant differences between treatments.

Table 3 illustrates the antimicrobial influence of the two *CPL* inclusion levels over 15 days of chilling on experimentally infected *LTL*-steaks with *Listeria monocytogens* and *Salmonella* compared to control and Sodium nitrite (NaNO_2_) pretreated *LTL*-steaks. Statistically, *Listeria monocytogens* and *Salmonella* growth curves were affected by the interaction of *CPL* inclusion levels and chilling storage length (*p* < 0.05). The *CPL* pretreatment levels, particularly *CPL0.5*, significantly inhibited *Listeria monocytogens* growth curves compared to control *LTL*-steaks from immediate inclusion at 1^st^ day to the end of chilling storage (*p* < 0.05). NaNO_2_ also showed comparable inhibitory influence on most checking-days of *Listeria monocytogens* growth curves compared to control. Both *CPL1* and *CPL0.5*, as well as NaNO_2_, had an immediate growth retardation effect on *Salmonella* growth curves until fifteen days post-treatment, keeping the *Salmonella* peak growth count below 2.76, 3.19, and 2 logs CFU/g, respectively, compared to 3.75 logs CFU/g for the control group. Notably, NaNO_2_ expressed a higher constant inhibitory influence than *CPL*.

**Table 3:** Antimicrobial influence of the two Cowpea-legumin (*CPL*) inclusion levels over 15 days of chilling on experimentally infected *Musculus longissimus thoracis* et *lumborum* (*LTL*) steaks with *Listeria monocytogens* and *Salmonella* compared to control and Na nitrite pretreated *LTL*-steaks

Pathogen	Groups	Pathogen growth throughout 15 days						SEM^*1*^	*P* value		
		1	3	6	9	12	15		Time	*CPL*^*1*^	*CPL* *Time
*Listeria monocytogens*	Control	3.18^Ad2^	3.54^Bd^	4.31^Ac^	4.53^Ac^	5.06^Ab^	6.80^Aa^	0.525	<0.001		
	*CPL1*^*1*^	2.65^Bb^	3.78^Ba^	2.85^Cb^	2.64^Cb^	2.65^Cb^	3.45^Ba^	0.200	<0.001		
	*CPL0.5*^*1*^	3.0^ABcd^	3.15^Cbc^	3.85^Ba^	3.23^Bbc^	2.72 ^Cd^	3.52^Bab^	0.162	<0.001		
	Nitrite	2.65^Bc^	4.73^Aa^	2.0^Dd^	2.0^Dd^	4.33^Bb^	2.0 ^Cd2^	0.512	<0.001		
	SEM	0.100	0.063	0.045	0.054	0.080	0.131				
	*p* value	0.025	<0.001	<0.001	<0.001	<0.001	<0.001		<0.001	<0.001	<0.001
*Salmonella enterica*	Control	3.1^Aab2^	3.02^b^	2.96^Ab^	3.25^Aab^	3.52^Aab^	3.75^Aa^	0.123	0.019		
	*CPL1*	2.69^Ba^	2.0 ^Bb^	2.0^Bb^	2.0^Bb^	2.0^Bb^	2.0^Bb^	0.116	<0.001		
	*CPL0.5*	2.98^ABb^	2.0^Bc^	2.0^Bc^	2.0^Bc^	3.2^Aab^	3.4^Aa^	0.281	<0.001		
	Nitrite	2.67^B^	2.0^B^	2.0^B^	2.0^B^	2.0^B^	2.0^B2^	0.112			
	SEM	0.079	0.015	0.015	0.007	0.077	0.120				
	*p* value	<0.001	<0.001	<0.001	<0.001	<0.001	<0.001		<0.001	<0.001	<0.001

^1^*CPL* cowpea-legumin, Control, *CPL1* 1 mg/g cowpea-legumin inclusion, *CPL0.5* 0.5 mg/g cowpea-legumin inclusion, *SEM* standard error of the mean, Nitrite, minced *LTL* blended with sodium nitrite at 150 PPM.

^2^Different small letters within the row show significant changes across chilling times (*p* < 0.05), while different capital letters within the column indicate significant differences between treatments.

## Discussion

The study’s objectives included determining the minimum antibacterial concentration of *CPL* against antibiotic-resistant/susceptible food-borne pathogens in vitro, as well as the effects of *CPL* on native spoilage flora, antioxidant stability, quality attributes, and growth curves of artificially inoculated food-borne pathogens in beef *LTL*-steaks. As evidenced by the resazurin turbidimetric microdilution experiment, 0.098 mg/mL *CPL* inhibited the growth of *Staphylococcus aureus*, which is both susceptible and resistant to antibiotics (ST72 and ST62). Antibiotic-susceptible *Salmonella* and *E. coli* were inhibited by the same minimum inhibitory concentration (MIC); however, antibiotic-resistant *E. coli* (EC55) and *Salmonella* (N7) were controlled by higher *CPL* MICs of 3.125 and 25 mg/mL, respectively. Although they employed a different method, the agar well diffusion test, earlier studies on legume proteins revealed that doses as low as 0.098 g/mL might suppress particular pathogens^20^. Additionally, 0.5 and 1 microgram of 7S and 11S globulins from red kidney beans (*Phaseolus vulgaris* L.) could inhibit antibiotic-resistant *K*. *pneumoniae* and *E*. *Coli*, according to a previous study on antibiotic-resistant Enterobacterales^22^. On the other hand, larger concentrations of different legume proteins (500 mg/mL) were added to milk; nonetheless, the MIC was ascertained in vivo right away without having to arrange for additional in-vitro testing^23^. Both current and previously published research support evidence that legume proteins, such as cowpea-legumin, have a promising antibacterial impact against MDR Gm-positive and Gm-negative bacteria and that they could be used as an alternative to routinely applied antibiotics to help combat antibiotic resistance^24^.

The findings showed that the inclusion of *CPL* from the first day was linked to a higher pH_U_ than the control group (Table 1). This finding could be explained by the proteinaceous and /or alkaline nature of cowpea-legumin (*CPL*)^21^; the dose-dependently higher pH of the two legumin-fortified *LTL*-steaks than control groups could support this assumption. The *CPL*-pretreated *LTL*-steaks produced evident spoilage pH after 15 days in both levels, whereas control *LTL*-steaks reached borderline pH after 6 days and apparent spoilage at 9 days (Table 1). Time-dependently raised pH can be attributed to the generation of spoilage metabolites, which are invariably produced with extended storage times due to oxidative and microbiological damage^25^. In this case, *CPL* was able to postpone these adverse interactions at every level.

The pH_U_ has been extensively employed as a potential meat quality predictor^26^. Higher WHC is always associated with higher pH^27^; however, in the present situation, there was no difference in WHC between the control and *CPL*-fortified groups, except for the sixth day, when the *LTL*-steak received *CPL* expressed higher WHC (Table 1). At this stage, the *CPL* addition produced this activity since the control group had a lower WHC but a higher pH.

Excessive purge loss from fresh meat correlates with losses in water, flavoring compounds, vitamins, and minerals, in addition to the monetary expenses associated with such products^28^. Both compared *LTL*-steak groups generated ascending purge loss curves with longer storage time and generated similar loss values except on the third-day post-treatment. The CL values of the two compared *LTL*-steak groups exhibited comparable findings throughout the trial, in line with purge loss (Table 1). The CL values of the control *LTL*-steaks did not vary throughout the six monitoring-points, while the *CPL1* inclusion showed a similar pattern to pH (Table 1). Previous research found that non-meat protein addition, such as leguminous Pea protein isolates, can increase the surface area of meat proteins that come into contact with water, improving water binding capacity and yield during cooking^18,29,30^. Excluding minor details, the tiny quantity of *CPL* used in the current investigation may be attributed to equivalent results of WHC, drip loss, and cooking loss between control and *CPL*-pretreated *LTL*-steaks. Furthermore, it has been noted that the 7S and 11S denature at temperatures of around 70°C and 90°C, respectively, which is necessary to acquire or improve technological capabilities^31^. This temperature may not have been attained under the current study cooking conditions, preventing *CPL* from experiencing sufficient structural changes during cooking, restricting their ability to interact with other components and their emulsification potential^32^.

Meat tenderness is frequently recognized as among the most essential quality features influencing customer acceptance of fresh meat^33^. The extent of structural myofibrillar protein integrity/degradation (proteolysis), the degree of sarcomere contraction or “sarcomere length,” and the composition and content of connective tissue are the key elements that determine meat tenderness^34^. The occurrence of meat tenderness is connected to the postmortem endogenous proteolytic degradation of muscle proteins. Changes in intramuscular circumstances, however, such as a decrease in pH, an increase in ionic strength, a decrease in reducing power, and the accumulation of reactive oxygen and nitrogen species, trigger this process^35^. Previous research has shown that the degree of meat tenderness and pH are correlated; as meat ages, high-pH meat tenderizes more quickly than low-pH meat. Here, however, the pH and WBSF outcomes demonstrate that the three proteolytic systems—cathepsin B, μ-calpain activities, and multicatalytic proteinase complex— perhaps were activated by *CPL*. Previously, suboptimal cathepsin B activity and intermediate μ-calpain activities were linked to intermediate pH_U_ (5.8 to 6.19)^36^. Additionally, *CPL* may counteract small heat shock proteins linked to harder meat at intermediate pH_U_, as observed in the control *LTL*-steaks^37^. Higher pH ultimate values (>6.2) in *CPL*-pretreated *LTL*-steaks than in control (intermediate pH_U_, 5.8 to 6.19) observed on the first day explain realistically that the addition of *CPL* provided a high pH (>6.2) environment in pretreated *LTL*-steaks that lasted until the twelve-day post-treatment, stimulating the interaction of the three components to promote tenderness process. This hypothesis^38^ could help to explain the overall low WBSF scores in *CPL*-pretreated *LTL*-steaks (Table 1). According to a previous report, the present tenderization capacity of *CPL* inclusion is classified with intermediated tenderness. A steak is deemed “tender” if its WBSF value falls between 3.36 and 4.28, and “intermediate tender” if its value falls between 4.3 and 5.4 kgf^39^.

In contrast to the clear dropping tendency in control *LTL*-steak’s L*, a* and chroma with longer storage duration, *CPL1* inclusion dropped L*, a* and chroma until the twelve-day compared to the last checking-day post-treatment (15), with a barely increasing trend. Meanwhile, *LTL*-steaks b* increased with *CPL1* inclusion and prolonged storage, except for the middle storage phase between the sixth- and ninth-days post-treatment, which did not differ from the control (Table 1). *CPL1*-pretreated *LTL*-steaks demonstrated less redness intensity than the control, defying the yellowness trend (Table 1). Prior studies revealed that the addition of plant protein, such as pea protein isolate, was associated with a dose-dependent increase in the b* value and a decrease in red products; nevertheless, there were differences in the L* values of meat products, ranging from low^3,40^ to high^30^. Here, *CPL*-pretreated *LTL*-steaks produced low, equivalent, and high L* values up until the end of storage (Table 1). The results of the most current study, which used soybean 11S globulin (SBG) on *LTL*-steaks, showed that most color parameters did not change from the control group, with the exception of yellowness and hue, which were higher in the *LTL*-steaks supplemented with SBG^41^. Previously, sensory analysis of minced beef supplemented with *CPL* demonstrated that the color was overall consistent during 4 °C storage^20^. The *CPL* inclusion, on the other hand, reduced myoglobin oxidation in a dose-dependent manner as compared to the decreasing redness pattern of control minced beef, which was potentially explained by higher oxidation to metmyoglobin levels in the control samples^20^. In shrimp, *CPL* spray could prevent oxidative rancidity and melanosis while keeping fresh color compared to control^42^.

Current oxidation results demonstrated that both *CPL* levels significantly and dose-dependently improved the oxidative stability of *LTL*-steaks compared to the control during the entire storage period (Table 1). Although different color evaluation methods were used^20^, the fewer red *LTL*-steaks obtained here could be attributed to the greater *CPL* content used (1 mg/g). However, the observation that the *CPL*-fortified *LTL*-steaks remained red until day twelve post-treatment when compared to the interrupted control group indicates that the antioxidant activity of *CPL* is sufficiently strong to stop the conversion of oxymyoglobin to metmyoglobin^43^ during this stage of the experiment. Previous research proposed two mechanisms for soybean protein antioxidant activity: protective and bioactive peptides. In the protective pathway, SBG binds noncovalently with highly oxidizable lipids and lipid-soluble nutrients such unsaturated fatty acids, enhancing oxidative stability and preventing oxidative degradation^44,45^. Second, the bioactive peptides produced by hydrolysis of SBG and boosted by the release of amino acids are thought to be responsible for the bioactive peptides’ high capacity to scavenge free radicals, reduce power, inhibit lipid peroxidation, and chelate metal ions^46,47^.

All *LTL*-steaks bacteriological indices were negatively influenced by *CPL*-pretreatment levels in a dose-dependent manner, with both low and elevated levels keeping APC below the spoilage level of 6 log CFU/g for 12 days (Table 2). Similarly, chickpea legumin supplementation (200 μg/mL) dramatically reduced APC, psychrotrophic count, and coliform counts in minced beef, improving the preservation period to 15 days^20^. Further, *CPL* exhibited a higher antibacterial effect on *Staphylococcus* counts than coliform (Table 2). Osman et al.^20^ also noticed that chickpea legumin supplementation had a higher inhibitory effect on *Staphylococcus aureus* (57%) than Gram-negative bacteria, *E*. *coli* (29%) and *P*. *aeruginosa* (51%) in minced beef. In terms of pH and microbiological assessments, *CPL* considerably increased the shelf-life of *LTL*-steaks at 4 °C from 6 days to 12 days. These results are consistent to previous findings of *CPL* on minced beef, where notable reduction in coliforms, psychrotrophs, and APC which remained below six log CFU/g even after 15 days of storage at 4 °C when treated with 50 and 100 µg/g of *CPL*^21^. The reasons for higher reduction activity in minced meat than in existing *LTL*-steaks may be due to the easily and uniformly dispersion of *CPL* powder when applied to minced meat rather than steak pieces weighing 60 g^48^. Previous investigations on extending the shelf-life of meat products revealed that the antimicrobial activity of powdered plant is less effective when used as a marinade and on whole meat as opposed to minced meat. This is because meat has the ability to act as a buffer, neutralizing the acidic marinade and causing lipophilic acids to dissociate^48,49^. Here, DW was added to enable even distribution of *CPL* on *LTL*-steaks with contact for half an hour, which appears to be ample time for meat to release buffering components, as demonstrated when meat pH is evaluated. Furthermore, the current *CPL* antimicrobial stability results were similar to earlier research on shrimp treated with 100 µg/ml and 200 µg/ml of *CPL*, which significantly reduced APC below 5.98–5.15 log CFU/g over 12 days, respectively^42^. The explanation for the higher reduction action in shrimp compared to the present steaks could be related to the 2 ± 0.5 °C chilling temperature. Controlling temperature and moisture is the foundation of practical strategies for preserving fresh meat^50^. Furthermore, other leguminous globulins, such as soybean glycinin (11S) in *LTL*-steaks^41^ and minced meat, and chickpea legumin (11S) in minced meat, shown equivalent and superior antibacterial activity than existing *CPL* in steak^20,23^. Previously, it was reported that cowpea seed protein globulins induced entire membrane degeneration, cell enlargement, vacuole development, and, finally, full lysis of cell components^21^. Similarly, swelling and disintegration of the bacterial cell membrane of both Gram-negative and Gram-positive bacteria have been reported as one of the antibacterial properties of soybean glycinin (11S) and chickpea legumin applied in beef and milk^20,23^.

Previous research, rather than using food matrix, concentrated on the invitro antimicrobial assessment of isolated legumes protein and peptides, soybean glycinin (11S) and chickpea legumin (11S)^20,23^, against food-borne pathogens such *Staphylococcus aureus*, *E*. *coli*, and *P*. *aeruginosa* on agar media. Here, however, the antibacterial impact and stability of the two *CPL* inclusion levels were estimated on experimentally contaminated *LTL*-steaks with *Listeria monocytogens* and *Salmonella enterica subspecies enterica* during 15 days of chilling versus control-negative beef and Na nitrite (NaNO_2_) pretreated meat (Table 3). The risk of contaminated meat and meat products arises from the vast distribution and several survival strategies of *Listeria monocytogenes* in raw materials, processing facilities, and retail environments, including vacuum (VP) conditions^51,52^. *Listeria monocytogenes* (*LM*) is a psychrotrophic bacterium that grows and adapts faster at 4 °C than at the greater chilling temperature, unlike mesophilic classified *Salmonella*^53^. This explains the considerable *LM* increase in control beef during refrigerated storage. However, cowpea-legumin pretreatment, especially at high levels, significantly decreased *Listeria monocytogens* growth curves from the first hour of incorporation to the end of chilling storage. The *LM* maximum count in *CPL*-treated meat ranged from 3.45 to 3.52 log CFU/g, while the *LM* maximum count in control beef was 6.80 log CFU/g, displaying a three-log decline (Table 3). Prior studies on the safety of meat products have established that synthetic nitrates and NaNO_2_ are crucial in reducing pathogens like *Clostridium botulinum*, *Listeria* spp., and *Salmonella* spp. They also provide a larger margin of safety and cannot be excluded from the hurdle technology during the process^54–56^. Both pathogen counts, especially those of *Salmonella*, were lower at all measured chilling-points in positive control *LTL*-steaks containing NaNO_2_ compared to control. For artificially inoculated pathogens in minced *LTL*-steaks, especially *LM*, neither natural *CPL* nor synthetic NaNO_2_ exhibited a uniform dropping curve despite their antibacterial interactions being equivalent in most of the checkpoints assessed. Pathogenic bacteria compete for AMPs with organisms for nutritional and ecological demands, resulting in greater flow through the cell wall. Through their hydrophobic and hydrophilic properties, AMPs can usually target many sites on pathogen plasma membranes and intracellular components, but they have limited cytotoxicity to mammals like as humans^57^. These advantages over existing antibiotics may explain the promising antibacterial influence of currently investigated *CPL* and their potential as medications in human infection and the meat sector compared to chemical preservatives.

The differences in shelf-lives observed between the current *CPL* and other studies conducted with either the same or different leguminous globulins provide insight into the factors that may affect the antibacterial efficacy and/or preservation stability of *CPL* when applied to fresh meat, as well as the most practical methods of application. The reason behind the limitation of *CPL’s* antimicrobial stability to only twelve chilling days, even with a higher pretreatment dose applied in the current study compared to previous studies, could be attributed to the native bacterial population and their defense mechanisms against natural antimicrobials, along with meat size, shape, pH, and temperature. Unfortunately, previous findings have shown that pathogenic bacteria, particularly resistant ones, can adapt to naturally occurring antimicrobials such peptides and AMP by developing pathways comparable to those of antimicrobial medicines. Modifications to the membrane or cell wall structure to obstruct one of the main AMP-based killing functions (i.e., to halt interaction with the negatively charged membrane), express resistance genes, sequester or inactivate AMP molecules, and remove them via efflux pumps or ABC transporters are some of these techniques^58^. Probable allergy is another factor that restricts the use of *CPL* as a fresh mat preservative. Although peas are not a priority allergenic food, Pis s1 and Pis s2 have been proposed as very rare possible allergens derived from vicilin and convicilin^59,60^.

In conclusion, *CPL* demonstrated promising antibacterial activity against *LTL*-steaks native flora, inoculated food-borne Gram-positive and -negative pathogens, and dose-dependent antioxidant activities that extended *LTL*-steaks freshness and improved tenderness by over twelve cooling days. *CPL* antibacterial efficacy and stability are controlled not only by dose and temperature, but also by matrix particle size, which has an impact on powder distribution and interaction. Moreover, the addition of *CPL* results in less red and yellow *LTL*-steaks in comparison to the control, but with extended storage times, the antioxidant capacity of *CPL* maintains color.

## Methods

### Experiment management and approval

Under the number BUFVM 10-06-2023, the Institutional Animal Care and Use Committee Research Ethics number (BUFVTM) of Benha University’s Faculty of Veterinary Medicine approved all the protocols used in this investigation. All related life science studies declarations are not applicable because the present study did not report results on humans or animals.

### Cowpea 11S globulin (legumin) protein extraction

The 7S globulin (vicilin) and 11S globulin (legumin) protein subunits were harvested from the defatted powder of cowpea seeds (*Vigna unguiculata* (L.) Walp.) using the previously published procedures with minor changes^21^. Centrifugation at 5000 × *g* for 10 min was carried out to extract the solubilized contents of 10 g of defatted cowpea seed powder dissolved in 150 mL of buffer (0.03 mol/L tris HCl at pH 8.5, 0.4 M NaCl, 10 mM -mercaptoethanol, 1 mM EDTA, 0.02% (w/v) NaN3). A 45°C water bath was used to stir the solution for 1 h. It was necessary to precipitate the 7S and 11S globulins using ammonium sulfate (50–65% and 65–85%, respectively). To precipitate 11S globulins, 65–85% ammonium sulfate was used. The precipitate was disseminated and solubilized as described above before being dialyzed against the same buffer for 48 h to remove the salts.

### The preparation of pathogens and inoculums

Two isolates of *Salmonella enterica* subspecies *enterica* (N7 from liver and N9 from minced meat), *Escherichia coli* (EC39, EC55, both from minced meat), and *Staphylococcus aureus* (ST62 from frozen minced meat and ST72v)—either multi-drug resistant (N7, EC55, and ST62) or non-resistant (EC39, N9, and ST72)—were applied for the microplate assessment of legumin’s antibacterial properties. All of the species were isolated from our earlier surveys, verified serologically by MALDI-TOF MS (VITEK®MS, database version 3, BioMerieux, France), and assessed quantitatively and phenotypically for antibiotic resistance using the VITEK®-2 system and the Kirby-Bauer disc diffusion method. Data on *Salmonella* antibiotic susceptibility, including molecular characterization, are available^12^; however, data on *Escherichia coli* and *Staphylococcus aureus* are still pending publication. For the challenge experiment, *Listeria monocytogenes* (ATTC 35152) were obtained from the Animal Health Research Institute (AHRI) in Dokki, Egypt.

### Cowpea legumin’s minimum inhibitory concentration (MIC)

Legumin’s MIC was ascertained using the turbidimetric microdilution method based on Resazurin^61^. A stock solution containing 50 mg/mL of legumin was generated using sterile distilled water. A 100 µl of this solution was applied to the first column of a 96-well plate, which was subsequently twice serially diluted on Columns 2–10. This resulted in a *CPL* concentration range of 50–0.1 mg/mL. After that, each well in Columns 1–10 received 50 µl of the previously diluted stock bacterial suspension at 6 log CFU/mL, resulting in a final concentration of 5–5.7 log CFU/mL. Column 12 held 100 µl of the medium broth (as a control to monitor sterility), and Column 11 held 100 µl of the diluted standardized inoculum. Following a 24 h incubation period at 37 °C, resazurin (0.015%) was introduced to each well (30 µl per well), and the wells were then incubated for an additional 2–4 h to determine any color changes. Columns with no color change (blue resazurin color stayed intact) at the end of the incubation period were assessed as being higher than the MIC value^61^. By immediately plating the contents of wells with concentrations greater than the MIC value, the minimum biocidal concentration (MBC) was ascertained^61^.

### Sample preparation and distribution

On the day of slaughter, fresh ribeye loin (*Musculus longissimus thoracis* et *lumborum*, *LTL*) from two beef carcasses were obtained from a local meat retailer and instantly delivered to the laboratory for investigation. *LTL* steaks weighing 60 ± 5 g (about 3 × 6 cm^2^ and 1.5-cm thick) were aseptically prepared from both sides of each carcass. Then were randomly allocated to one of three treatments: no *CPL* addition (control), fortification with 1 mg/g of *CPL* (*CPL1*), and fortification with 0.5 mg/g of *CPL* (*CPL0.5*). The allocation was carefully manipulated to ensure that the two carcasses *LTL* steaks were included in the three treatments over the six checking chilling days. The individual treatment involved forty-eight steaks divided into two replicates, each consisting of twenty-four pieces. Each replicate was allocated over six checking days, with four steaks per day. *LTL*-steaks of the *CPL1* group were directly supplied with 1 mg/g *CPL* powder, then 100 mL of sterile distilled water (DW) was added into the zippered package to achieve equal powder dispersion across all steaks, followed by a moderate rotation. After 30 min, the steaks were placed on a sterilized steel sieve to dry. The same procedures were employed for the control group, which just used Sterile DW, but 0.5 mg/g legumin was used in the *CPL0.5* treated *LTL*-steaks. The *LTL*-steaks were then bagged and refrigerated in the cooling incubator (Binder KB, BINDER GmbH, Tuttlingen, Germany) at 4 ± 0.1 °C. The physicochemical quality of the *longissimus* was evaluated on days 0, 3, 6, 9, 12, and 15. Water-holding capacity (WHC), purge loss, cooking loss, Warner-Bratzler Shear Force (WBSF), and instrumental color of *LTL*-steaks were only compared between the Control and *CPL1* groups. The shelf-life, pH, and antioxidant stability of *LTL*-steaks, on the other hand, were evaluated in three separate groups: control (DW, 1^st^), *CPL1* (1 mg/g *CPL*, 2^nd^), and *CPL0.5* (0.5 mg/g *CPL*, 3^rd^). The antibacterial activity and stability of *CPL* in minced *LTL*-steaks against artificially inoculated food-borne pathogens were evaluated using four groups: control, *CPL1*, and *CPL0.5*, as well as a fourth group of minced *LTL*-steaks blended with sodium nitrite at 150 PPM as a positive control for synthetic preservatives.

### Physicochemical evaluation of longissimus attributes

The Physicochemical properties of *LTL*-steaks were assessed by applying previously published methods. These characteristics included color parameters (L*, Lightness; a*, Redness; b*, Yellowness; *C*, Chroma; and *h°*, Hue angle) as well as pH, WHC, purge loss, cooking loss (CL), Warner-Bratzler shear force (WBSF)^62^.

#### pH analysis

The *LTL* samples designated for microbiological and pH assessment were individually tenfold diluted with sterile distilled water and analyzed for pH using pH-meter electrodes (Jenway 3510 pH-meter, Cole-Parmer, Staffordshire, United Kingdom) at each checking point for each holding container. At room temperature, the pH meter was calibrated using three different pH levels (10, 4, and 7) in conjunction with a temperature metal probe. After the microbiological analysis was done, the pH was assessed.

#### Water-holding capacity estimation

With minor modifications, the low-speed centrifugation techniques outlined by Honikel and Hamm (1994) were used to assess the water-holding capacity (WHC). In brief, a sample of 5 g of intact *LTL*-steak was centrifuged for 20 min at 10.000 × *g* and 5 °C in a 15 mL falcon tube filled with glass beads. The *LTL*-steak was then removed with forceps, dried with filter paper, and weighed again. The WHC was expressed as the percentage of the weight difference between the *LTL*-steaks before and after the centrifugation.

#### Purge loss estimation

The purging loss at each checking point is estimated using the percentage of *LTL*-steak weight loss from the initial weight recorded on the first day of chilling (0, 3, 6, 9, 12, and 15 day)^63^.

#### Cooking loss estimation

On each checking day, three samples were subsampled from chilled *LTL* duplicates to assess cooking loss and shear force. Shear force blocks were randomly assigned to two cook batches based on the replication number. The *LTL* samples, previously weighed, were individually placed in plastic thermotolerant bags with thin walls and heated for 30 min at 80 °C in a water bath. They were then chilled with tap water to room temperature, cooled in an ice bath to 5 °C, dried, and weighed again. CL is the percentage difference between the raw and cooked weights^63^.

#### Warner-Bratzler Shear Force (WBSF) analysis

Using cooked *LTL*-steak samples, the WBSF was determined using an Instron 3343 Universal Test Device Mono column (Norwood, MA, USA). Perpendicular to the direction of the fiber, the cores of every *M*. *longissimus* were severed from the anterior end. The WBSF value, expressed in kilogram force (kgf), was the average measurement of six cores from each steak^64^.

#### Instrumental color estimation

In the raw *LTL*-steaks, three colors were assessed using the CR-410 chromometer (Konica Minolta Sensing INC., Osaka, Japan). Chroma Meter set to observer angle of 2°, aperture size of 8.0 mm with closed cone, L*, a*, b* color space, and illuminant D65. Measurements were taken throughout the cut surface of the *LTL*-steak after it had bloomed for 30 min. Before measurement, the chromameter was calibrated using a reference white tile. These color values were then used to estimate color saturation as (*Hue* angle (*h*˚) = arctg *b* */*a* *) and color intensity as (C =(a*^^2^ + b*^^2^) ^^0.5^). Increased chroma values signify heightened saturation in the primary hue of the sample. Higher hue angle (or color intensity) values, on the other hand, correspond to less red meat^65^. Six measurements were averaged out for each group.

### Microbiological assessment of chilled M. longissimus

Furthermore, the aerobic plate count (APC), coliform count, lactic acid bacteria count, and staphylococcal count of *LTL*-steak generated from compared groups were assessed over 15 days (1, 3, 6, 9, 12 and 15) in programable cooling incubator (Binder KB, BINDER GmbH, Tuttlingen, Germany) at 4 °C.

#### Determination of aerobic plate count

The aerobic plate count (APC) in *LTL*-steaks was assessed the same way as for ground beef products^66^. Each sample was produced as a 10% homogenate, then serially diluted ten times, with 1 mL of each dilution inserted into two separate sterile Petri dishes. After that, the solidified inoculation plates were incubated for 24 h at 37 °C^67^.

#### Determination of coliform count

One mL of previously prepared tenfold dilutions was inoculated into two independent sterile Petri dishes of Violet red bile agar at 37 °C for coliform enumeration in *LTL*-steak^68^.

#### Determination of Staphylococcus count

*Staphylococcus* counts in *LTL*-steaks were determined triple using the surface-plating method on the Baird Parker agar plate, as previously published for milk^69^. One milliliter of each of the previously made serial dilutions was distributed using a sterile disposable spreader. The plates were left upright in the incubator for approximately 10 min, or 1 h until the agar absorbed the inoculums. After that, the inoculated were inverted and incubated at 37 °C for 48 h.

#### Lactic acid bacteria count

Man, Rogosa, and Sharpe medium Agar (MRS, HiMedia, USA) was used to determine *LAB*, and samples were cultured anaerobically at 30 °C for 72 h^66^.

### Cowpea-legumin in-vivo antibacterial activity and stability

*CPL* was tested in-vivo for antibacterial activity and stability against *L*. *monocytogenes* and *Salmonella enterica* subspecies *enterica* (N7) in minced *LTL*. As a positive control for synthetic preservatives, another set of minced *LTL*-steaks containing sodium nitrite (NaNO_2_) at 150 PPM was prepared. The minced *LTL*-steaks were categorized into four groups: control, *CPL1*, *CPL0.5*, and NaNO_2_-treated. Minced *LTL*-steaks of *CPL1* and *CPL0.5* groups were blended directly with cowpea-legumin at 1 mg/g and 0.5 mg/g, respectively, whereas the fourth group was mixed thoroughly with 150 PPM of NaNO_2_. To obtain a final inoculated pathogen initial level of 3 log CFU/g in minced *LTL*, a fresh working culture of *L*. *monocytogenes* and *Salmonella enterica* (N7) from an overnight culture at 37 °C was serially adjusted to 5 log CFU/mL and injected into the four minced *LTL* groups at a rate of 2 mL/100 g^66^. Then, 10 g of each group was transferred to a sterile glass bottle (125 mL with rubber closure). Three bottles (three replicates) from each of the four groups were randomly assigned to the six checkpoints (1, 3-, 6-, 9-, 12- and 15-day post-treatment) and then stored at 4 ± 0.1 °C in the incubator (Binder, BINDER GmbH, Tuttlingen, Germany). To undertake in-vivo microbiological investigations, 10 g of the spiked minced *LTL*-steak was homogenized for one min in a sterile stomacher bag using 90 mL of sterile distilled water (Stomacher 400 R, Seward, UK). Once the homogenization and decimal dilutions were completed, 100 μL was applied to Hektoen enteric agar plates (Condalab, Spain) and Oxford agar (supplied with selective Oxford supplement, Oxoid, Hampshire, United Kingdom) to count *Salmonella* spp. and *L*. *monocytogenes*, respectively. The colonies exhibiting a certain morphology were counted 24 h after the inoculation plates were incubated at 37 ± 0.2 °C.

### Malondialdehyde estimation

Using HPLC (Agilent HP 1200 series system, USA) and the guidelines provided by refs. ^70,71^, malondialdehyde (MDA) levels in homogenized *LTL*-steaks were determined. Using an ice-cold homogenizer, a 10% sample homogenate (w/v) and ice-cold 0.1 M Tris-HCl pH 7.4 were created (Glas-Col, USA). The homogenate was centrifuged for 15 min at 2000 × *g* at 4 °C to remove debris. In addition, 1 mM of stock standard MDA solution was prepared by dissolving 25 μL of 1,1,3,3 tetra-ethoxy-propane (TEP) in 100 mL of water. After that, the 20 nmol/mL working standard was made by dissolving 1 mL of TEP stock solution in 50 mL of 1% sulfuric acid for 2 h at room temperature, which was then diluted with 1% sulfuric acid to yield a final standard concentration of 1.25 nmol/mL for total MDA analysis. The results were expressed as nM/g.

### Statistical analyses

The data analysis was conducted using SPSS Version 22. (SPSS Inc. Chicago, IL, USA). The impact of treatments (control, *CPL1*), chilling periods (1-, 3-, 6-, 9-, 12-, and 15-day), and their interaction on *LTL*-steaks quality metrics was studied using general linear mixed models (GLM). *LTL*-steaks were considered random, whereas treatments and chilling storage time were fixed variables. Similar statistical techniques were used for WBSF and cooking loss; however, the random model included cooking batches. To assess *LTL*-steaks microbiological, antioxidant, and pH characteristics, models included fixed effects for treatments (control, *CPL1*, and *CPL0.5*) and chilling storage durations (1, 3, 6, 9, 12, and 15 days), random terms for *LTL*-steaks, and interaction effects (treatment × chilling day). In-vivo *CPL* antibacterial activity was examined using fixed effects of treatment (control, *CPL1*, *CPL0.5*, and nitrite) and chilling storage durations (1, 3, 6, 9, 12, and 15 days), random terms for minced *LTL*-steaks bottles, and an interaction term between treatment and chilling day. The results are shown with their means and the overall standard errors of the means. The statistical model employed Tukey’s b multiple comparison test to quantify the effects of *CPL* and their levels, as well as the impacts of NaNO_2_ compared to control, and to assess unique monitoring point averages within the same group. Significant differences were determined with a *p*-value of less than 0.05.

## Data Availability

All materials described in the manuscript, including all relevant raw data, were included as main and supplementary tables and figures. The datasets generated during and analyzed during the current study are available from the corresponding author upon reasonable request.
